# Water activity in liquid food systems: A molecular scale interpretation

**DOI:** 10.1016/j.foodchem.2017.06.046

**Published:** 2017-12-15

**Authors:** Andrew J. Maneffa, Richard Stenner, Avtar S. Matharu, James H. Clark, Nobuyuki Matubayasi, Seishi Shimizu

**Affiliations:** a Green Chemistry Centre of Excellence, Department of Chemistry, University of York, Heslington, York YO10 5DD, United Kingdom; b York Structural Biology Laboratory, Department of Chemistry, University of York, Heslington, York YO10 5DD, United Kingdom; c Division of Chemical Engineering, Graduate School of Engineering Science, Osaka University, Toyonaka, Osaka 560-8531, Japan; d Elements Strategy Initiative for Catalysts and Batteries, Kyoto University, Katsura, Kyoto 615-8520, Japan

**Keywords:** Kirkwood-Buff, KB, Water activity, Water structure, Statistical thermodynamics, Kirkwood-Buff theory, Sugar, Polyol

## Abstract

Water activity has historically been and continues to be recognised as a key concept in the area of food science. Despite its ubiquitous utilisation, it still appears as though there is confusion concerning its molecular basis, even within simple, single component solutions. Here, by close examination of the well-known Norrish equation and subsequent application of a rigorous statistical theory, we are able to shed light on such an origin. Our findings highlight the importance of solute-solute interactions thus questioning traditional, empirically based “free water” and “water structure” hypotheses. Conversely, they support the theory of “solute hydration and clustering” which advocates the interplay of solute-solute and solute-water interactions but crucially, they do so in a manner which is free of any estimations and approximations.

## Highlights

• Confusion concerning the molecular basis of water activity still persists.
• Rigorous statistical thermodynamics revealed the molecular basis of water activity.
• The Norrish constant can be interpreted in terms of interactions in aqueous solutions.
• Water activity is driven by interplay of solute-solute/solute-water interactions.

## 1 Introduction

Following its introduction over 60 years ago (Scott, 1953), the application of “water activity” has become omnipresent within food science and related disciplines. It serves as a useful indicator of the microbiological stability of foodstuffs and food-related systems, much better than mere water content (Scott, 1957). Water activity also plays an important role on the sensorial properties of foodstuffs such as aroma, taste and texture as well as on their chemical and biological reactivity (e.g., lipid oxidation and non-/enzymatic activity) (Labuza & Rahman, 2007). Despite serious criticisms on the utility of water activity as a fundamental descriptor of water-related phenomena in food and related systems, it continues to be widely used to this day as a tool for product development and quality control across multiple areas of the food industry (Slade & Levine, 1991). However, what “water activity” really is on a microscopic scale is still a matter of controversy, which we focus on exclusively in this paper. There are the following three different views co-existing in the literature:

1. the fraction of “free or available water” due to the presence of the “bound water” around solutes (sugars or polyols);
2. the measure of “water structure” formation or the “ordering” of water in the presence of the solutes (sugars or polyols), and;
3. the measure of “hydration water” which comes from the stoichiometric clustering/bindings models of water and solutes.

The **“Free water” hypothesis** (Scott, 1953) advocates the use of water activity “as a measure of the availability of water” in an aqueous medium (i.e., solution). The popular view, water activity as water “availability”, may have been originated from this seminal paper, giving rise to the interpretation that water activity is a measure of water freedom (i.e., boundness). In any case, this view suggests that water activity of an aqueous solution is fundamentally reflected by only the nature of the water interactions occurring within it. Despite the best attempts to remedy such over-simplification (Altunakar & Labuza, 2008), the interpretation of water activity in terms of “free water” still persists (Carareto et al., 2010, Frosch et al., 2010, Guine et al., 2015).

The **“Water structure” hypothesis** advocates that the addition of certain species into solution either has: i. the effect of increasing or decreasing the activity of water on account of weakening, or; ii. strengthening the ‘structure’ or ‘ordering’ of the water network surrounding the added solute. This hypothesis appears to be an extension of the classical view that solutes can either promote or interfere with the hydrogen bonding network of water in solution (i.e., act as “structure makers” or “structure breakers”) (Frank and Evans, 1945, Frank and Franks, 1968). Such an interpretation suggests that water activity should therefore predominantly be a function of water-water interaction, with increased interaction (i.e., more order or structure) leading to a reduction in water activity (Caurie, 2005, Dutkiewicz and Jakubowska, 2002). Even though the contribution from solute-solute interaction is acknowledged to be present in water activity this contribution is considered to be small and negligible (Sato and Miyawaki, 2008, Sone et al., 2015).

The **Hydration number and stoichiometric clustering hypothesis** is based upon a stoichiometric binding reaction model of solute hydration. Scatchard related water activity to ‘hydration number’ i.e., number of ‘bound’ water molecules (Scatchard, 1921b). This relationship was employed later by Stokes and Robinson to understand the origin of water activity in aqueous non-electrolyte solutions (Stokes & Robinson, 1966). Further extension of this approach to aqueous sucrose solutions accounts for both hydration and solute clustering modelled in terms of a series of stepwise stoichiometric reactions (Gharsallaoui et al., 2008, Starzak and Mathlouthi, 2006, VanHook, 1987). This approach has revealed competition between hydration and clustering, i.e., i. sucrose hydration lowers water activity, and; ii. sucrose clustering raises water activity by increasing the effective mole fraction of water.

Thus, we see that there have been three different hypotheses on the origin of the water activity. Are these hypotheses equivalent or contradictory? To the best of our knowledge this question has not been suitably answered. Instead of constructing another thermodynamic model of water activity, the aim of the current work is to establish what water activity really means on a molecular scale. To do so, we employ the first principles of statistical thermodynamics without any models or approximations (Shimizu, 2004, Shimizu and Matubayasi, 2014b) unlike the stoichiometric approach to clustering and association which depends on a number of model assumptions (such as the size of clusters and equilibrium constants) (Funke et al., 1989, Gharsallaoui et al., 2008, Guggenheim, 1952, Scatchard, 1921a, Schönert, 1986a, Schönert, 1986b, Starzak and Mathlouthi, 2006, Starzak et al., 2000, Stokes and Robinson, 1966, VanHook, 1987), and cannot describe interactions within solutions and mixtures, which are weak, non-specific and dynamic in nature, in a realistic manner (Shimizu, 2004, Shimizu, 2013, Shimizu and Matubayasi, 2014b, Shimizu et al., 2017). The rigorous statistical thermodynamic approach, on the contrary, has revealed the molecular picture at odds with most of the previous hypotheses on the role of solutes on solution thermodynamics and solubility (Shimizu et al., 2017).

Indeed, Shimizu has demonstrated that the gradient of water activity with respect to solute mole fraction is determined by the compensation between solute-solute and solute-water interactions (Shimizu, 2013). This is contradictory to the free water and water structure hypotheses, but is consistent with the solute clustering models on the molecular origin of “water activity”. However, this study focused on the *gradient* of water activity instead of the water activity itself. Hence, herein, the first full statistical thermodynamic clarification of how solute-water and solute-solute interactions contribute to the water activity itself is reported.

## 2 A statistical thermodynamic basis of water activity and the Norrish equation

To reveal the molecular basis of water activity, we combine insights from food chemistry with respect to statistical thermodynamics. In recent years, the application of an exact, model-free approach, i.e., the Kirkwood-Buff (KB) theory of solutions (Ben-Naim, 2006, Chitra and Smith, 2002, Kirkwood and Buff, 1951, Shimizu, 2004), has proven to be a powerful tool for improving the microscopic understanding of various liquid food systems including gelatin (Shimizu & Matubayasi, 2014a), tofu (Shimizu et al., 2017) and aqueous sucrose solutions (Shimizu, 2013). Here we clarify the molecular basis of water activity within this rigorous theoretical framework.

In food science, water activity of liquid food has been modelled successfully by the Norrish equation (Norrish, 1966)

(1) $$a_{w}=x_{w}e^{{\mathrm{Kx}}_{s}^{2}}$$

where K is referred to as the Norrish constant and the system consists of two components (water and solute). Eq. (1) is reported to fit the water activity in high to medium water content and has been used in the context of food science as a useful fitting equation (Baeza et al., 2010, Fysun et al., 2015). Note that Eq. (1) is identical to the one-parameter Margules equation where K is often represented as α.

In statistical thermodynamics, Eq. (1) can be derived rigorously from first principles (see Appendix A). In the framework of the Kirkwood-Buff theory (Chitra and Smith, 2002, Kirkwood and Buff, 1951, Shimizu, 2004, Shimizu and Matubayasi, 2014b), the following three contributions to the Norrish constant can be identified

(2) $$K=\frac{1}{2V_{w}^{\infty }}\left(G_{\mathrm{ww}}^{\infty }+G_{\mathrm{ss}}^{\infty }-2G_{\mathrm{sw}}^{\infty }\right)$$

where $G_{\mathrm{ww}}$, $G_{\mathrm{ss}}$ and $G_{\mathrm{sw}}$ respectively signify the water-water, solute-solute and solute-water KB integral (KBI), $V_{w}$ is the partial molar volume of water, and the superscript ∞ signifies at the infinite dilution of solute. KBI is defined as

(3) $$G_{\mathrm{ij}}=4\pi \int \mathrm{dr}\;r^{2}[g_{\mathrm{ij}}(r)-1]$$

where $g_{\mathrm{ij}}(r)$ refers to the radial distribution function between the species i and j. KBIs are the quantitative measure of affinity between species in solution. Note that Eq. (2) has been derived at the infinite dilution of solute. Here the difference between self- ($G_{\mathrm{ww}}^{\infty }$ and $G_{\mathrm{ss}}^{\infty }$) and mutual interaction ($G_{\mathrm{sw}}^{\infty }$) has been identified, for the first time, as the molecular-level interpretation of the Norrish constant. Note that $G_{\mathrm{ww}}^{\infty }+G_{\mathrm{ss}}^{\infty }-2G_{\mathrm{sw}}^{\infty }$ has been shown previously to be the key for the *gradient* of water activity with respect to solute concentration (Shimizu, 2013). Previous works on the application of the Kirkwood-Buff theory to binary mixtures (Ben-Naim, 2006, Chitra and Smith, 2002, Shimizu, 2013) have employed the general and rigorous theoretical expressions applicable to all concentration ranges, while the present work, aiming at clarifying the meaning of the Norrish constant, focuses on the infinite dilution limit of the solute. The theoretical expressions used in the present paper can be derived directly from the previous, more general theory, as has been demonstrated in Appendices A and B, Appendices A and B.

The statistical thermodynamic derivation of the Norrish equation shows that, strictly speaking, it is accurate only at the infinite dilution of solutes. However, in practice, the Norrish equation can be used over much wider range of solute concentrations with relatively good accuracy. This means that the water-water, solute-water and solute-solute KBIs at infinite solute dilution are crucial factors that determine the water activity up to moderate solute concentrations (*ca.* 60 wt%, *ca.* 5–10 mol dm^−3^ or a mole fraction of 0.1–0.2).

To quantify the relative contributions of water-water, solute-water and solute-solute KBIs to the Norrish constant, additional experimental data are indispensable. Volumetric data will indeed complement the Norrish constant and lead to the determination of individual KBIs through the use of the following well-known relationships (Shimizu, 2004, Shimizu and Matubayasi, 2014b):

(4) $$G_{\mathrm{sw}}^{\infty }=-V_{s}^{\infty }+\mathrm{RT}\kappa_{T}^{\infty }$$

(5) $$G_{\mathrm{ww}}^{\infty }=-V_{w}^{\infty }+\mathrm{RT}\kappa_{T}^{\infty }$$

where $V_{s}^{\infty }$, $V_{w}^{\infty }$ and $\kappa_{T}^{\infty }$ respectively signify the partial molar volume of solute, water (at infinite dilution of solutes) and isothermal compressibility of pure water. Hence the three KBIs can be determined via three experimental data (Eqs. (2), (4), (5)).

## 3 Molecular basis of water activity: both solute-solute and solute-water interactions contribute to the Norrish constant

### 3.1 The Norrish constant as a competition between solute-water and solute-solute interactions

The combination of the Norrish equation with rigorous thermodynamic theory reveals an entirely different molecular-based make-up of the Norrish constant, thereby leading to a reconsideration of the molecular basis of water activity.

**Fig. 1:**
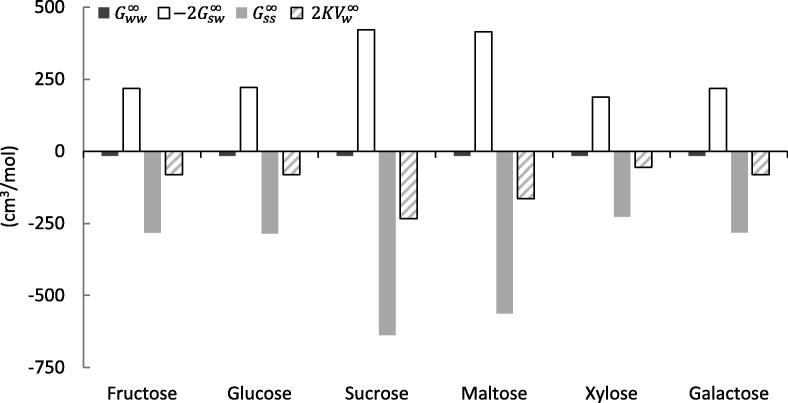
Molecular origin of the Norrish constant *K*. Comparison between KBIs $G_{\mathrm{ww}}^{\infty }$, $-2G_{\mathrm{sw}}^{\infty }$ and $G_{\mathrm{ss}}^{\infty }$ and the corresponding $2{\mathrm{KV}}_{w}^{\infty }=G_{\mathrm{ww}}^{\infty }+G_{\mathrm{ss}}^{\infty }-2G_{\mathrm{sw}}^{\infty }$ for various binary aqueous sugar solutions.

**Fig. 2:**
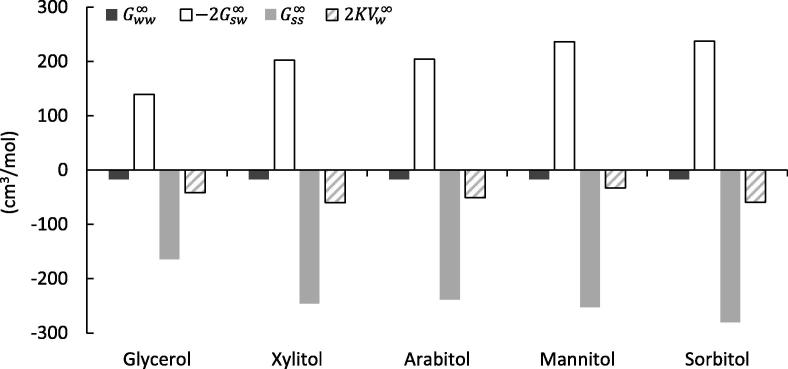
Molecular origin of the Norrish constant *K*. Comparison between KBIs $G_{\mathrm{ww}}^{\infty }$, $-2G_{\mathrm{sw}}^{\infty }$ and $G_{\mathrm{ss}}^{\infty }$ and the corresponding $2{\mathrm{KV}}_{w}^{\infty }=G_{\mathrm{ww}}^{\infty }+G_{\mathrm{ss}}^{\infty }-2G_{\mathrm{sw}}^{\infty }$ for various binary aqueous polyol solutions.

The Norrish constant is made up of water-water, solute-water and solute-solute interactions, expressed via the KBIs. In the following, their relative contributions to the Norrish constant are quantified. Figs. 1 and 2 highlight the contribution of each of the KBI terms for binary aqueous solutions of various sugars and polyols respectively, to the overall value of the Norrish constant (data is summarised in Table 1). In the case of the solutes studied in the present work, it can be seen that *both* $G_{\mathrm{ss}}^{\infty }$ and $G_{\mathrm{sw}}^{\infty }$ make significant contributions to the Norrish constant with $G_{ss}^{\infty }$ the more dominant of the pair. As has been mentioned previously, this conclusion is markedly different to the “free water” and “water structure” hypotheses which were outlined in Section 1.

**Table 1:** Values of the Norrish constant, K and individual Kirkwood-Buff integrals ($G_{\mathrm{sw}}^{\infty }$, $G_{\mathrm{ww}}^{\infty }$ and $G_{\mathrm{ss}}^{\infty }$) for each species used in this study (cm^3^ mol^−1^). $G_{\mathrm{sw}}^{\infty }$ and $G_{\mathrm{ww}}^{\infty }$ were calculated according to Eqs. (4), (5) respectively and using values of *V_w_*^∞^ = 18.1 cm^3^ mol^−1^ and *κ_T_*^∞^ = 4.53 × 10^−10^ Pa^−1^. Note that all calculations are strictly only valid at 298 K.

Species	*K*	$V_{s}^{\infty }$^c^	$G_{\mathrm{sw}}^{\infty }$	$G_{\mathrm{ww}}^{\infty }$	$G_{\mathrm{ss}}^{\infty }$
*Sugars*					
Fructose	−2.25^a^	110	−109	−16.9	−283
Glucose	−2.25^a^	112	−111	−16.9	−286
Sucrose	−6.47^a^	212	−210	−16.9	−638
Maltose	−4.54^a^	209	−208	−16.9	−562
Xylose	−1.54^a^	95.4	−94.3	−16.9	−227
Galactose	−2.24^a^	110	−109	−16.9	−282

*Polyols*					
Glycerol	−1.16^a^	71.0	−69.8	−16.9	−165
Xylitol	−1.66^a^	102	−101	−16.9	−246
Arabitol	−1.41^b^	103	−102	−16.9	−238
Mannitol	−0.91^a^	119	−118	−16.9	−252
Sorbitol	−1.65^a^	120	−119	−16.9	−280

a Taken from Taoukis and Richardson (2007).

b Taken from Rahman (1995).

c Taken from Cabani, Gianni, Mollica, and Lepori (1981).

The following has emerged from our analyses:

1. Solute-solute interaction drives up the Norrish constant
2. Solute-water interaction drives down the Norrish constant
3. The Norrish constant, much smaller than 1 and 2, is the result of compensation between 1 and 2.

These insights have been obtained directly from experimental data and the principles of statistical thermodynamics without any model assumptions. Because of the fundamental nature of the above insights, we are now in the position to examine the accuracy and validity of the previous hypotheses on the molecular basis of water activity, summarised in Section 1.

With respect to the **“Free water” hypothesis**, if the “bound water” (i.e., water which is not “free”) in solution can be interpreted as $G_{\mathrm{sw}}^{\infty }$ in the context of our theory, then $G_{\mathrm{sw}}^{\infty }$ is, in almost all cases, a less dominant contributor to the Norrish constant than $G_{\mathrm{ss}}^{\infty }$. Similarly, if it is simply “free” or “bound” water which are the primary origins of water activity, we may also expect an interplay between $G_{\mathrm{ww}}^{\infty }$ and $G_{\mathrm{sw}}^{\infty }$ to have a substantial influence on K. This however, is not observed. Furthermore, the signs of K and $G_{\mathrm{sw}}^{\infty }$ are opposite.

In contrast to the **“Water structure” hypothesis**, it is the compensation between solute-solute and solute-water interaction rather than solely the properties of solvents which drives up the Norrish constant. In the context of the Kirkwood-Buff theory, if “water structure” or “ordering” is the origin of water activity then we should expect the water-water interaction term, $G_{\mathrm{ww}}^{\infty }$ to have a significant influence on K. In fact, what we actually observe is that $G_{\mathrm{ww}}^{\infty }$ is small relative to $G_{\mathrm{sw}}^{\infty }$ and especially $G_{\mathrm{ss}}^{\infty }$ and thus, has negligible effect on the Norrish constant. This conclusion is consistent with previous criticisms of ‘water structure’ by KB theory (Shimizu et al., 2017). Note that our results also highlight that inter-solute interactions ($G_{\mathrm{ss}}^{\infty }$) are a key contributor to water activity in contrast to their supposedly negligible role as advocated in both “free water” and “water structure” hypotheses.

With respect to the **“Hydration” and clustering hypothesis**, our results have identified that the activity coefficient of water is a result of a compensation of two large contributions; i. $G_{\mathrm{sw}}^{\infty }$ which drives down the water activity, and; ii. $G_{\mathrm{ss}}^{\infty }$ which drives up the water activity. This is contradictory to the earlier theories that attempted to explain water activity solely from hydration (i.e., solute-water interaction) but is consistent with the later development of the models that incorporated the effect of solute (sucrose) clustering. Intuitively speaking, water activity is driven up by solute clustering as it increases the effective mole fraction of water (Gharsallaoui et al., 2008, Starzak and Mathlouthi, 2006, Starzak et al., 2000, VanHook, 1987). Previous models have reached such a conclusion through a series of equilibrium constant estimations that was crucial for modelling the water activity over a wide range of concentrations. Our interpretation, unlike previous models, is based only on the first principles of statistical thermodynamics, which supports the insights from the clustering models.

### 3.2 Why the Norrish equation describes water activity beyond infinite dilution

The Norrish equation (Eq. (1)), according to rigorous statistical thermodynamics, has been shown to be valid only at infinite solute dilution. In contrast to this theoretical foundation, the Norrish equation has been used to fit the water activity far beyond infinite dilution. It has been reported that the Norrish equation holds well until *ca*. 60% w/w% for various nonelectrolytes, including sucrose (Baeza et al., 2010). Why is the Norrish equation applicable beyond infinite dilution? This can be understood by a comparison between the “Norrish approximation” and the rigorous KB theory. To do so, we need to employ the general formalism of the KB theory for a binary solution mixture, applicable to the entire concentration range, which has been well-established (Ben-Naim, 2006, Chitra and Smith, 2002) and has been applied for the analysis of aqueous sucrose solutions (Shimizu, 2013). We have shown in Appendix B that the Norrish equation is accurate when

(6) $$2K=\frac{1}{x_{w}}\frac{c_{w}(G_{\mathrm{ww}}+G_{\mathrm{ss}}-2G_{\mathrm{sw}})}{1+x_{s}c_{w}(G_{\mathrm{ww}}+G_{\mathrm{ss}}-2G_{\mathrm{sw}})}$$

behaves virtually as a constant over a wide concentration range, where $x_{w}$ is the mole fraction of water, $x_{s}$ is for solute, $c_{w}$ is the molarity of water, and the KBIs in the r.h.s. are in principle dependent on the concentration.

Based upon previous analysis of sucrose (Shimizu, 2013) using the fitting model of Starzak and Mathlouthi (2006), Fig. 3 shows the change of the r.h.s. of Eq. (6) against sucrose w/w%, which demonstrate its increase is very slow, thereby demonstrating the accuracy of the Norrish approximation based on its constancy. Note that the r.h.s. of Eq. (6) computed from the water activity model (−16.4) (Starzak and Mathlouthi, 2006), is closer to the value (−14.8) calculated from the two-parameter Margules (Miyawaki, Saito, Matsuo, & Nakamura, 1997) rather than the one from the Norrish constant from literature (2*K* = −12.9) (Taoukis & Richardson, 2007), which may come from the fact that the Norrish K represents an average of the r.h.s. of Eq. (6) over a wide sucrose concentration range. What is important here is that the weak sucrose concentration dependence of K comes from $G_{\mathrm{ww}}+G_{\mathrm{ss}}-2G_{\mathrm{sw}}$, whose sucrose concentration dependence has been shown to be considerably weaker than the much stronger concentration dependencies of $G_{\mathrm{ss}}$ and $G_{\mathrm{sw}}$ (Shimizu, 2013). This suggests that the presence of compensation between $G_{\mathrm{ss}}$ and $G_{\mathrm{sw}}$ that is responsible for the near-constancy of K. Whether this mechanism holds true for other solutes will be investigated in our future publications.

**Fig. 3:**
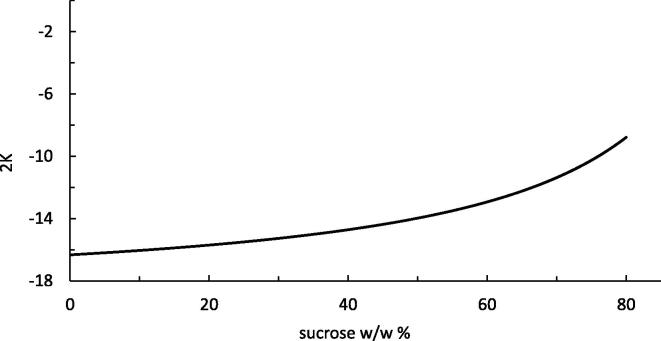
Accuracy of the Norrish equation beyond infinite sucrose dilution, demonstrated by the slow change of the r.h.s. of Eq. (6) with respect to sucrose concentration.

The effective radii of sucrose and water can provide a rough justification of the KBIs and the Norrish constant. Assuming water and sucrose as spheres, the sucrose-sucrose and water-water co-volumes, $-G_{\mathrm{ss}}$ and $-G_{\mathrm{ww}}$, correspond to the effective radii of 3.2 and 0.94 Å for water and sucrose, which are smaller than their commonly-quoted hard-sphere radii, because the peaks of the correlation functions contribute negatively to co-volumes (Shimizu, 2013). Using these effective radii, $-G_{\mathrm{sw}}$ can be estimated to be 174 cm^3^ mol^−1^, which is only *ca.* 15% smaller than the value in Table 1. The reasonable success of this rough estimation suggests that there seems to be a simple volumetric mechanism at work, which may determine much of the KBIs and the Norrish constant and may be behind the slow change of 2K in Fig. 3. A more quantitative treatment is possible only by an explicit treatment of intermolecular interactions via molecular simulations.

## 4 Conclusion

Due to the lack of a theoretical foundation, the molecular origin of water activity in liquid food systems has long been obscure. There have been three hypotheses in the literature, yet even whether they are consistent or contradictory has remained unanswered.

To address this historical question, we have combined the wisdom of food science with the rigorous statistical thermodynamics. Based upon the Kirkwood-Buff theory of solutions, we have identified the origin of the Norrish constant, i.e., water-water, solute-water and solute-solute interactions, amongst which water-water is a minor contribution. The Norrish constant is a product of compensating contributions from the solute-solute and solute-water interactions. Solute-solute interaction drives up the Norrish constant while the solute-water interaction is in the opposite direction to the Norrish constant.

In contrast to the previous work, solute-solute interaction has quantitatively been identified as a crucial contributor to the water activity. This conclusion based on a rigorous theory is inconsistent with two traditional hypotheses of water activity origin; “free water” and “water structure”, both of which are primarily built upon solely empirical data and argue that solute-solute interplay is effectively negligible. The compensation between solute-solute and water-solvent interactions clarified by our rigorous theory corroborates more recent solute hydration and clustering models from the first principles of statistical thermodynamics.

## Appendix A

We derive the statistical thermodynamic expression of the Norrish constant based on a well-known result from the KB theory (Ben-Naim, 2006, Shimizu, 2013), namely

(A1) $${\left(\frac{\partial \mu_{w}}{\partial x_{s}}\right)}_{T\text{,}P}=-\frac{\mathrm{RT}}{x_{w}}\frac{1}{1+x_{s}c_{w}(G_{\mathrm{ww}}+G_{\mathrm{ss}}-2G_{\mathrm{sw}})}$$

where $c_{w}$ is the molar concentration of water. Note that $G_{\mathrm{ww}}+G_{\mathrm{ss}}-2G_{\mathrm{sw}}$ plays a crucial role in Eq. (A1) (Shimizu, 2013). At $x_{s}\to 0$, the r.h.s. of Eq. (A1) can be expanded as:

(A2) $${\left(\frac{\partial \mu_{w}}{\partial x_{s}}\right)}_{T\text{,}P}\approx -\frac{\mathrm{RT}}{x_{w}}\left[1-x_{s}\frac{G_{\mathrm{ww}}^{\infty }+G_{\mathrm{ss}}^{\infty }-2G_{\mathrm{sw}}^{\infty }}{V_{w}^{\infty }}\right]$$

where $V_{w}^{\infty }$ is the partial molar volume of pure water. It is useful to rewrite Eq. (A2) in terms of the activity coefficient of water, as

(A3) $${\left(\frac{\partial \ln\gamma_{w}}{\partial x_{s}}\right)}_{T\text{,}P}=\frac{1}{\mathrm{RT}}{\left(\frac{\partial \mu_{w}}{\partial x_{s}}\right)}_{T\text{,}P}+\frac{1}{x_{w}}\approx x_{s}\frac{G_{\mathrm{ww}}^{\infty }+G_{\mathrm{ss}}^{\infty }-2G_{\mathrm{sw}}^{\infty }}{V_{w}^{\infty }}$$

Comparing Eq. (A3) with the Norrish equation, $\ln\gamma_{w}={\mathrm{Kx}}_{s}^{2}$, we obtain the following final form:

$$K=\frac{1}{2}\frac{G_{\mathrm{ww}}^{\infty }+G_{\mathrm{ss}}^{\infty }-2G_{\mathrm{sw}}^{\infty }}{V_{w}^{\infty }}$$

Note that the physical meaning of the infinite-dilution KB integral, $G_{\mathrm{ss}}^{\infty }$, can be understood directly from its relationship to the second virial coefficient, $B_{2}$, via $G_{\mathrm{ss}}^{\infty }=-2B_{2}$ (Ben-Naim, 2006).

## Appendix B

The Norrish equation can fit water activity far beyond infinite solute dilution. To explain why this is possible, let us start by rewriting Eq. (A1) as

(B1) $${\left(\frac{\partial \ln\gamma_{w}}{\partial x_{s}}\right)}_{T\text{,}P}=\frac{1}{x_{w}}\frac{x_{s}c_{w}(G_{\mathrm{ww}}+G_{\mathrm{ss}}-2G_{\mathrm{sw}})}{1+x_{s}c_{w}(G_{\mathrm{ww}}+G_{\mathrm{ss}}-2G_{\mathrm{sw}})}$$

The rigorous expression (Eq. (B1)) should be compared with the approximation used in deriving the Norrish equation. Hence the task is to show the accuracy of

(B2) $$\frac{1}{x_{w}}\frac{c_{w}(G_{\mathrm{ww}}+G_{\mathrm{ss}}-2G_{\mathrm{sw}})}{1+x_{s}c_{w}(G_{\mathrm{ww}}+G_{\mathrm{ss}}-2G_{\mathrm{sw}})}\approx \frac{G_{\mathrm{ww}}^{\infty }+G_{\mathrm{ss}}^{\infty }-2G_{\mathrm{sw}}^{\infty }}{V_{w}^{\infty }}$$
